# Suppression of the Lycopene Cyclase Gene Causes Downregulation of Ascorbate Peroxidase Activity and Decreased Glutathione Pool Size, Leading to H_2_O_2_ Accumulation in *Euglena gracilis*

**DOI:** 10.3389/fpls.2021.786208

**Published:** 2021-12-03

**Authors:** Shun Tamaki, Ryosuke Sato, Yuki Koshitsuka, Masashi Asahina, Yutaka Kodama, Takahiro Ishikawa, Tomoko Shinomura

**Affiliations:** ^1^Department of Biosciences, School of Science and Engineering, Teikyo University, Tochigi, Japan; ^2^Advanced Instrumental Analysis Center, Teikyo University, Tochigi, Japan; ^3^Center for Bioscience Research and Education, Utsunomiya University, Tochigi, Japan; ^4^Institute of Agricultural and Life Sciences, Academic Assembly, Shimane University, Matsue, Japan

**Keywords:** *Euglena gracilis*, carotenoid, lycopene cyclase, ascorbate-glutathione cycle, reactive oxygen species, antioxidant, RNAi

## Abstract

Carotenoids are photosynthetic pigments and hydrophobic antioxidants that are necessary for the survival of photosynthetic organisms, including the microalga *Euglena gracilis*. In the present study, we identified an uncharacterized gene encoding the *E. gracilis* β-carotene synthetic enzyme lycopene cyclase (EgLCY) and discovered a relationship between EgLCY-mediated carotenoid synthesis and the reactive oxygen species (ROS) scavenging system ascorbate-glutathione cycle. The *EgLCY* cDNA sequence was obtained *via* homology searching *E. gracilis* transcriptome data. An enzyme assay using *Escherichia coli* demonstrated that EgLCY converts lycopene to β-carotene. *E. gracilis* treated with *EgLCY* double-stranded RNA (dsRNA) produced colorless cells with hypertrophic appearance, inhibited growth, and marked decrease in carotenoid and chlorophyll content, suggesting that EgLCY is essential for the synthesis of β-carotene and downstream carotenoids, which are abundant and physiologically functional. In *EgLCY* dsRNA-treated cells, the ascorbate-glutathione cycle, composed of ascorbate peroxidase (APX), dehydroascorbate reductase (DHAR), monodehydroascorbate reductase (MDAR), and glutathione reductase (GR), was unusually modulated; APX and GR activities significantly decreased, whereas DHAR and MDAR activities increased. Ascorbate content was significantly increased and glutathione content significantly decreased in *EgLCY* dsRNA-treated cells and was correlated with their recycling enzyme activities. Fluorescent imaging demonstrated that *EgLCY* dsRNA-treated cells accumulated higher levels of H_2_O_2_ compared to wild-type cells. Taken together, this study revealed that EgLCY-mediated synthesis of β-carotene and downstream carotenoid species upregulates APX activity and increases glutathione pool size for H_2_O_2_ scavenging. Our study suggests a possible relationship between carotenoid synthesis and the ascorbate-glutathione cycle for ROS scavenging in *E. gracilis*.

## Introduction

**Figure 1:**
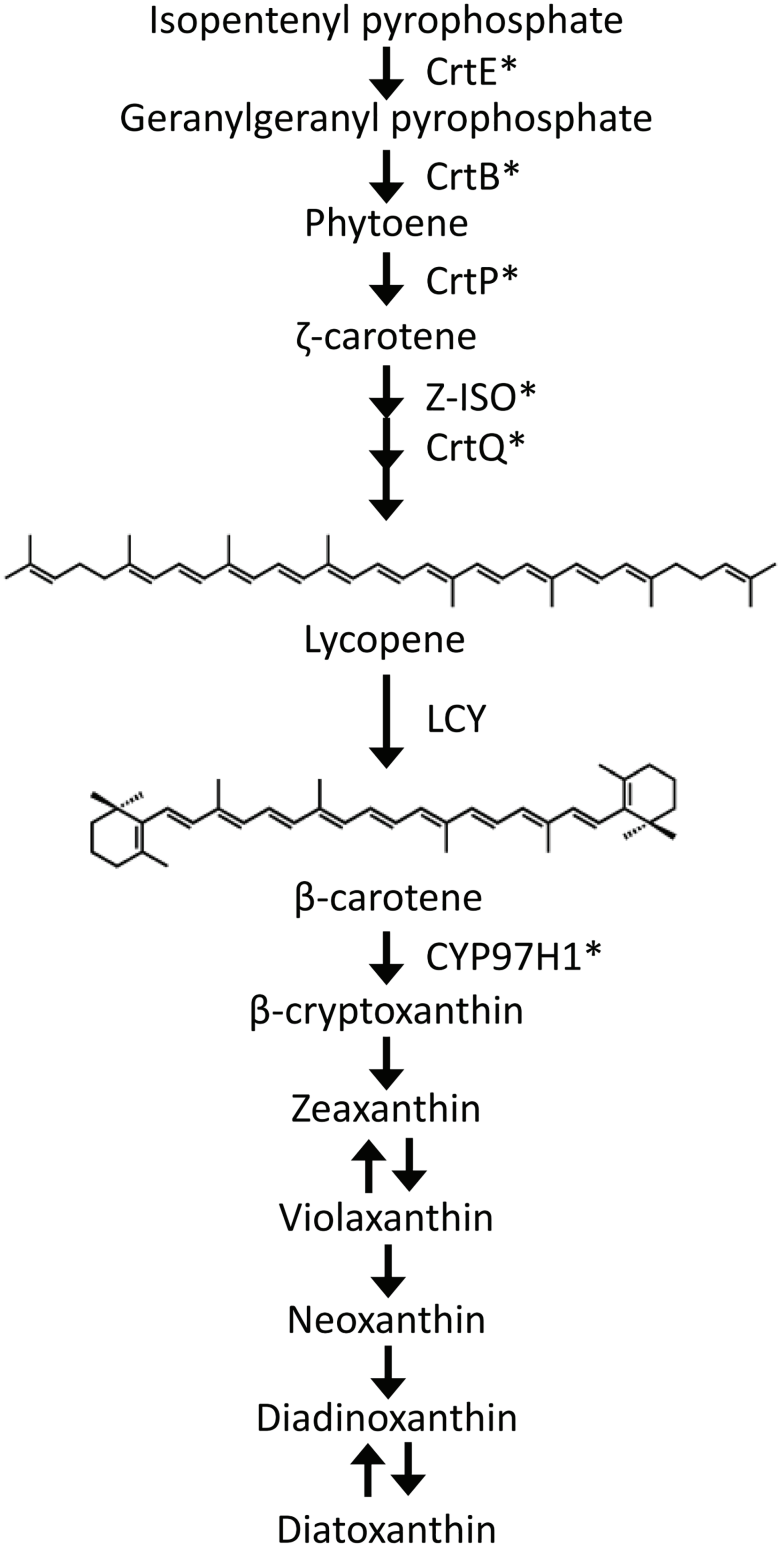
Schematic diagram of the carotenoid synthetic pathway in *Euglena gracilis*. CrtE, geranylgeranyl pyrophosphate synthase; CrtB, phytoene synthase; CrtP, phytoene desaturases; Z-ISO, ζ-carotene isomerase; CrtQ, ζ-carotene desaturase; LCY, lycopene cyclase; and CYP97H1, β-carotene hydroxylase. ^*^functionally characterized in previous studies.

Carotenoids are isoprenoid compounds with C_40_ backbones and are naturally widespread pigments that range in absorbance from yellow to red. The unicellular microalga *Euglena gracilis* contains diadinoxanthin, diatoxanthin, neoxanthin, and β-carotene as the major carotenoid species (Kato et al., 2017). We have genetically and biochemically characterized several carotenoid synthetic genes in *E. gracilis* to understand the carotenoid synthesis pathway (reviewed in Tamaki et al., 2021). In the *E. gracilis* carotenoid synthetic pathway (Figure 1), the most upstream carotenoid species, phytoene, is synthesized from isopentenyl pyrophosphate by geranylgeranyl pyrophosphate synthase (CrtE) and phytoene synthase (CrtB; Kato et al., 2016). Phytoene is then desaturated and isomerized to lycopene by phytoene desaturases (CrtP1 and CrtP2), ζ-carotene desaturase (CrtQ), and ζ-carotene isomerase (Z-ISO; Kato et al., 2019; Sugiyama et al., 2020). Lycopene is cyclized to β-carotene by lycopene cyclase (LCY). Subsequently, β-carotene is hydroxylated by CYP97H1 (Tamaki et al., 2019) and converted to neoxanthin, diadinoxanthin, and diatoxanthin through several uncharacterized steps. Our recent study demonstrated that carotenoid content was associated with cold and high light stress response in *E. gracilis* (Kato et al., 2019). This suggests that carotenoids are critical in supporting environmental stress tolerance by this alga. In contrast, LCY is directly involved in β-carotene synthesis, and β-carotene is synthesized furthest upstream among the four major *E. gracilis* carotenoid species. We recently demonstrated that increased β-carotene accumulation alleviates photoinhibition under high light stress by promoting PSII electron transfer during photosynthesis (Tanno et al., 2020). Moreover, it has been reported that β-carotene synthesis is photo-induced not only in wild-type *E. gracilis*, but also in chloroplast-deficient strain (Dolphin, 1970). Therefore, functional analysis of LCY is essential for understanding the stress tolerance mechanism(s) in *E. gracilis*.

LCY homologs are known to be involved in carotenoid synthesis. Lycopene β-cyclases (LCYB, CrtL, CruA, CruP, and CrtY) that form β-rings at the ends of lycopene are present in plants, eukaryotic algae, cyanobacteria, and bacteria (Cunningham et al., 1994, 1996; Schnurr et al., 1996; Maresca et al., 2007). Lycopene ε-cyclases (LCYE) that form the ε-ring during α-carotene synthesis are present in plants and green algae (Cunningham et al., 1996). The lycopene β-cyclase gene has not yet been identified in *E. gracilis*, and the lycopene ε-cyclase gene is predicted to be absent due to the absence of α-carotene and its derivatives, such as lutein, in this organism.

In many organisms, including animals, plants, and algae, carotenoids act as reactive oxygen species (ROS) scavengers (Gammone et al., 2015; Foyer, 2018; Tamaki et al., 2021). ROS, such as hydrogen peroxide (H_2_O_2_), superoxide radicals, hydroxyl radicals, and singlet oxygen are byproducts of aerobic cellular processes, including photosynthesis and respiration, and cause oxidative damage to cells upon excessive accumulation. The superoxide radical is generated by the reduction of molecular oxygen in the photosynthetic electron transport chain and the respiratory chain. It is then converted to H_2_O_2_ *via* the reaction with superoxide dismutase (SOD). Singlet oxygen is generated by transferring the energy of the photoexcited chlorophyll to molecular oxygen (Foyer, 2018; Smirnoff and Arnaud, 2019). Many organisms have developed a variety of ROS scavenging systems to avoid ROS toxicity (Apel and Hirt, 2004). In addition to carotenoids that mainly scavenge singlet oxygen and do not react with H_2_O_2_ and superoxide radicals, other antioxidants, such as ascorbate and glutathione, directly react with various ROS molecules and scavenge them in many organisms including plants and algae (Foyer, 2018; Smirnoff and Arnaud, 2019; Tamaki et al., 2021). In photosynthetic organisms, including plants and algae, ascorbate peroxidase (APX) detoxifies H_2_O_2_ by using reduced ascorbate as an electron donor. Ascorbate and glutathione are oxidized during this cycle, but their oxidized forms are regenerated by the ascorbate-glutathione cycle consisting of dehydroascorbate reductase (DHAR), monodehydroascorbate reductase (MDAR), and glutathione reductase (GR). These antioxidants and enzymes are physiologically important as a defense system against ROS-derived oxidative stress (Mittler et al., 2004; Tamaki et al., 2021). *E. gracilis* accumulates both ascorbate and glutathione at high levels and contains ascorbate-glutathione cycle enzymes that have been well characterized biochemically, whereas catalase is absent in this alga (Shigeoka et al., 1980, 1987a,b; Ishikawa et al., 2017). APX has also been reported to play a role in cellular H_2_O_2_ metabolism in *E. gracilis* (Ishikawa et al., 2010). Although *APX* gene expression is induced post-transcriptionally when *E. gracilis* cells are transferred from dark to light conditions, this induction is inhibited by treatment with the carotenoid synthesis inhibitor norflurazon (Madhusudhan et al., 2003). This finding suggests an interaction between carotenoid synthesis and induction of the ascorbate-glutathione cycle, including APX, in this alga; however, strong evidence of the functional relationship between carotenoid synthesis and the ascorbate-glutathione cycle has not been convincingly established in photosynthetic organisms.

To further elucidate the carotenoid synthetic pathway and define the role of carotenoid synthesis in the induction of the ascorbate-glutathione cycle in *E. gracilis*, we identified the *E. gracilis LCY* (*EgLCY*) gene *via* homology searching and functionally characterized it. An *in vivo* enzyme assay using *Escherichia coli* demonstrated that EgLCY is a lycopene β-cyclase. Moreover, the effects of *EgLCY* suppression on the ascorbate-glutathione cycle and cellular H_2_O_2_ accumulation were investigated. Our results suggested that EgLCY-mediated synthesis of β-carotene and downstream carotenoid species causes upregulated APX activity and increased glutathione pool, resulting in protection against H_2_O_2_ accumulation in *E. gracilis*.

## Materials and Methods

### Culture Conditions of *E. gracilis*

*Euglena gracilis* Klebs (strain Z) were grown in 100 ml of Cramer-Myers (CM) medium (Cramer and Myers, 1952) containing 0.1% (v/v) ethanol as a carbon source, at an initial cell concentration of 3 × 10^3^ cells ml^−1^. The mixture was kept in a 300 ml conical flask under continuous light conditions (40 μmolm^−2^ s^−1^) using fluorescent lamps FL40SEX-N-HG (NEC lighting, Tokyo, Japan) at 25°C with rotary shaking (90 rpm) using a Double Shaker NR-30 (Taitec, Aichi, Japan). The light intensity was measured using a 3664 optical power meter (Hioki, Nagano, Japan).

### Cloning and Heterologous Expression of the *EgLCY* Gene in *E. coli*

Total RNA was isolated from *E. gracilis* using the Cica geneus RNA Prep Kit (for Plant) (Kanto Chemical, Tokyo, Japan), in accordance with the manufacturer’s instructions. First-strand cDNA was synthesized using a PrimeScript RT reagent kit with gDNA Eraser (Takara Bio, Shiga, Japan), in accordance with the manufacturer’s instructions. The open reading frame of *EgLCY* was amplified from first-strand cDNAs using the EgLCY-F and EgLCY-R primer set. All primers used in this study are listed in Supplementary Table 1. The amplified DNA fragments were ligated into the pMD20-T vector (Takara Bio) to check for PCR errors. *EgLCY* DNA fragments for the In-Fusion reaction were amplified from the obtained *EgLCY* clone using the EgLCY-InFusion-F and EgLCY-InFusion-R primer set. The expression vector pETDuet-1 DNA (Merck, NJ, United States) was digested with NcoI and reacted with amplified DNA fragments using the In-Fusion HD Cloning Kit (Takara Bio). The constructed plasmid was designated as pET-EgLCY. The pET-EgLCY was co-transformed into *E. coli* BL21(DE3) (New England Biolabs, MA, United States) with pACCRT-EIB (Misawa et al., 1995), which facilitates the accumulation of lycopene by heterologous expression of lycopene biosynthetic genes *crtE*, *crtB*, and phytoene desaturase *crtI* from *Pantoea ananatis* in host cells.

The lycopene-accumulating *E. coli* cells with pETDuet-1 empty vector or pET-EgLCY were grown in 3 ml of LB medium containing 50 μgml^−1^ ampicillin and 30 μgml^−1^ chloramphenicol. After overnight culture at 37°C, the cultures were inoculated into 2 × YT medium (1.6% tryptone, 1% yeast extract, and 0.5% sodium chloride) with the same concentrations of antibiotics and grown to an OD_600_ of 0.5. IPTG was added to a concentration of 0.5 mM, and the cultures were incubated for 48 h at 21°C. The cultured medium was centrifuged, and *E. coli* cell precipitate was stored at −60°C.

### Extraction and Analysis of Carotenoids From *E. coli*

Under dim light, carotenoids were extracted from *E. coli* cells with 1 ml of acetone. After centrifugation, the extracts were dried using a rotary evaporator. Carotenoid extracts were dissolved in 1 ml of ethyl acetate and filtered using a Columngard-LCR_13_ (Merck) prior to HPLC analysis. Fifteen microliters of extracts were analyzed in an HPLC system equipped with a PEGASIL ODS SP100 column (6 mM × 150 mM, 5 μm particles, Senshu Scientific, Tokyo, Japan). The mobile phase was acetonitrile/methanol/tetrahydrofuran (58:35:7, v/v/v) at a flow rate of 0.8 mlmin^−1^. Absorbance spectra (250–700 nm, 1.2-nm resolution) and retention times were recorded for 50 min using a Photodiode Array Detector SPD-M20A (Shimadzu, Kyoto, Japan).

### RNAi Experiments

An approximately 500-bp *EgLCY* partial cDNA template with the T7 RNA polymerase promoter was amplified using the EgLCY-RNAi-F and EgLCY-RNAi-R primer set (Supplementary Table 1). The double-stranded RNA (dsRNA) was synthesized from this PCR product using a MEGAscript RNAi Kit (Thermo Fisher Scientific, MA, United States), in accordance with the manufacturer’s instructions. The electroporatic introduction of *EgLCY* gene dsRNA into *E. gracilis* cells was performed following the method described in our previous study (Tamaki et al., 2019). Aliquots of the culture were collected daily, and cell density was determined using a microscope equipped with a plankton counting chamber. The cultured medium was centrifuged, and the *E. gracilis* cell precipitate was stored at −60°C.

### Semi-Quantitative RT-PCR Analysis

Total RNA was isolated from six-day-old *E. gracilis* cells, into which dsRNA was introduced, and then, first-strand cDNA was synthesized as described above. Suppression of targeted gene expression was confirmed by semi-quantitative RT-PCR analysis using the EgLCY-sqRT-PCR-F and EgLCY-sqRT-PCR-R primer set (Supplementary Table 1). The *E. gracilis* Actin (*EgActin*) gene was chosen as a control, using the EgActin-sqRT-PCR-F and EgActin-sqRT-PCR-R primers (Supplementary Table 1). The EgAPX, EgGR, and EgDHAR genes were amplified using the EgAPX-sqRT-PCR-F and EgAPX-sqRT-PCR-R, EgGR-sqRT-PCR-F and EgGR-sqRT-PCR-R, and EgDHAR-sqRT-PCR-F and EgDHAR-sqRT-PCR-R, respectively. PCR amplification conditions consisted of 22–35cycles at 98°C for 15s, 55°C for 30s, and 68°C for 30s. The PCR products were analyzed by gel electrophoresis on a 1% agarose gel. The amount of each amplified DNA fragment was corrected using the *EgActin* PCR products.

### Chlorophyll and Carotenoid Measurements

Chlorophyll and carotenoid extraction and measurements were performed following the method described in our previous study (Tamaki et al., 2019).

### Enzyme Assays

*Euglena gracilis* (2 × 10^7^ cells) was suspended in 500 μl of ice-cold buffer (50 mM potassium phosphate buffer, pH 7.0) and disrupted by sonication. The cell lysate was centrifuged at 100,000 × g at 4°C for 60 min, and the supernatant was used for each enzyme assay. For the APX activity assay, buffer was replaced with 50 mM potassium phosphate, pH 7.0, containing 1 mM EDTA and 1 mM ascorbate.

The APX activity assay was performed following the method described in our previous study (Tamaki et al., 2014). GR activity was measured as the decrease in absorbance at 340 nm due to NADPH oxidation. The reaction mixture contained 1 mM EDTA, 0.2 mM NADPH, and 1 mM oxidized glutathione in 50 mM potassium phosphate buffer, pH 8.2, in a final volume of 1 ml. DHAR activity was measured as the increase in absorbance at 265 nm due to dehydroascorbate reduction. The reaction mixture contained 2.5 mM reduced glutathione and 0.2 mM dehydroascorbate in 50 mM potassium phosphate buffer, pH 7.0, in a final volume of 1 ml. MDAR activity was measured as the decrease in absorbance at 340 nm due to NADPH oxidation. The reaction mixture contained 1 mM ascorbate, 0.2 mM NADPH, and 0.2U ascorbate oxidase in 50 mM potassium phosphate buffer (pH 7.0) in a final volume of 1 ml. SOD activity was measured using a SOD assay kit-WST (Dojindo Molecular Technologies, Inc., Kumamoto, Japan) in accordance with the manufacturer’s instructions.

### Antioxidant Quantification

To determine ascorbate, 5 × 10^7^ *E. gracilis* cells were suspended in 600 μl of ice-cold 2% metaphosphoric acid and disrupted by sonication. The cell lysate was centrifuged at 15,000 rpm at 4°C for 15 min. Ten microliters of extracts were analyzed in an HPLC system equipped with a Mightysil RP-18 GP analytical column (4.6 mM × 150 mM, 5 μm particles, Kanto Chemical, Tokyo, Japan) and guard column (4.6 mM × 5 mM, 5 μm particles, Kanto Chemical). The mobile phase was 1% metaphosphoric acid at a flow rate of 0.5 mlmin^−1^. Ascorbate was detected at 245 nm using a Photodiode Array Detector SPD-M20A. The reduced ascorbate concentrations were calculated from the peak area of the HPLC chromatogram using the calibration curve of the authentic ascorbate standard. Total ascorbate was measured after reducing the oxidized form by incubating with 35 mM tris (2-carboxyethyl) phosphine hydrochloride at 4°C for 3 h. The contents of oxidized ascorbate were calculated as the difference between total and reduced ascorbate.

To determine glutathione, 5 × 10^7^ *E. gracilis* cells were suspended in 1 ml of ice-cold 0.2 M HCl and disrupted by sonication. The cell lysate was centrifuged at 15,000 rpm at 4°C for 10 min. One hundred microliters of 0.2 M potassium phosphate buffer, pH 5.6, was added to an aliquot of 0.5 ml of supernatant and vortexed. The pH of the extracts was adjusted to 4–5 by adding small volumes of 0.2 M NaOH. Total glutathione was determined using an enzymatic recycling assay based on GR. The reaction mixture contained 5 mM EDTA, 0.5 mM NADPH, 0.6 mM 5,5′ dithiobis-(2 nitrobenzoic acid), and cell extract in 100 mM potassium phosphate buffer, pH 7.5, in a final volume of 1 ml. The reaction was initiated by the addition of 1 unit of GR (Oriental Yeast, Tokyo, Japan), and the absorbance at 412 nm was monitored for 2.5 min. The total glutathione content was calculated from the increase in absorbance using the calibration curve of the authentic glutathione standard. The oxidized glutathione was selectively determined by assaying samples in which glutathione was masked by pretreatment with 2-vinylpyrimidine. Two microliters of 2-vinylpyrimidine was added to 200 μl of extracts and incubated at room temperature for 30 min after vortexing. After that, it was assayed as described above. The difference between the total glutathione and oxidized glutathione contents was presented as the reduced glutathione content.

### Visualization of Cellular H_2_O_2_ Levels

Intracellular H_2_O_2_ levels in *E. gracilis* cells were detected by staining with the fluorescent reagent BES-H_2_O_2_-Ac (FUJIFILM Wako Pure Chemical, Osaka, Japan). BES-H_2_O_2_-Ac was dissolved in dimethyl sulfoxide at a concentration of 1 mM as a stock solution. To begin, 1 × 10^6^ *E. gracilis* cells were collected by centrifugation, and the medium was completely removed. Cells were resuspended in 500 μl of medium that contained 2% dimethyl sulfoxide and 20 μM BES-H_2_O_2_-Ac and incubated at room temperature for 30 min in the dark. Cells without BES-H_2_O_2_-Ac were incubated in medium that contained 2% dimethyl sulfoxide. After incubation, cells were centrifuged and washed twice with 1 ml of fresh medium. Cells were resuspended in 50 μl of fresh medium and immediately observed using a confocal laser microscope. The fluorescent images were obtained under excitation at 488 nm using a TCS SP8X (Leica, Wetzlar, Germany) equipped with a highly flexible pulsed white-light laser (WLL). For imaging in green wavelength regions, a 485 nm laser (21% of WLL) was employed. The BES-H_2_O_2_-Ac emission spectra was detected at 490–580 nm using a hybrid detector. Chlorophyll autofluorescence was eliminated by time-gated fluorescence imaging (gate-on time: 0.3–12.0nsec; Kodama, 2016). The fluorescence images were acquired at 100 Hz (100 lines s^−1^) with a 3 × line average.

## Results

### Primary Structure and Functional Analysis of EgLCY

The cDNA sequence of the putative *EgLCY* gene (GenBank accession number: LC590220) was obtained *via* a tblastn search of *E. gracilis* transcriptome data (GenBank accession number: GDJR00000000.1; Yoshida et al., 2016) using *Arabidopsis thaliana* LCYB (GenBank accession number: U50739) as the query. The cDNA sequence generated by this search in *E. gracilis* had a spliced leader sequence, which is a characteristic short sequence that is normally transferred to the 5′ end of pre-mature mRNAs by trans-splicing in *E. gracilis* (Tessier et al., 1991). This indicates that recovered sequence is a full-length cDNA.

The EgLCY amino acid sequence shares 30–36% identity and 44–50% similarity with lycopene β-cyclases from *A. thaliana*, *Solanum lycopersicum*, *Phaeodactylum tricornutum*, and *Synechococcus elongatus* PCC 7942. The amino acid sequence alignment and phylogenetic tree of LCY proteins from *E. gracilis* and other photosynthetic organisms and bacteria are shown in Supplementary Figures 1, 2, respectively. The FAD/NADPH-binding site and cyclase motifs of LCY proteins are conserved in EgLCY and lycopene β-cyclases from other organisms (Cunningham et al., 1996). In contrast, EgLCY was phylogenetically separated from other lycopene cyclases (LCYB, LCYE, CrtL, CruA, CruP, and CrtY) from photosynthetic organisms and bacteria.

**Figure 2:**
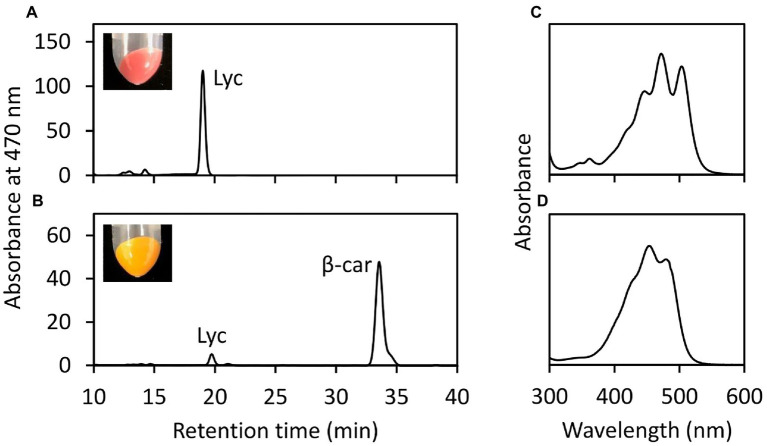
*In vivo* EgLCY enzyme assay in *E. coli* cells. HPLC chromatogram (470 nm) of carotenoid extracts of lycopene-accumulating *E. coli* cells transformed with pETDuet-1 empty vector **(A)** and pET-EgLCY plasmid **(B)**. Insets are photographic images of *E. coli* pellets. Absorbance spectra of detected peaks of lycopene **(C)** and β-carotene **(D)**. Lyc, lycopene; β-car, β-carotene.

To evaluate the enzyme activity of EgLCY, the *EgLCY* cDNA was heterologously expressed using a T7 expression vector in *E. coli* cells. *E. coli* cells co-transformed with pACCRT-EIB, which expresses the lycopene synthetic genes *crtE*, *crtB*, and *crtI* from *Pantoea ananatis*, and a pETDuet-1 empty vector was red in color and produced large amounts of lycopene. These lycopene-accumulating *E. coli* cells became orange in color and accumulated β-carotene as the main carotenoid when the *EgLCY* gene was co-expressed (Figure 2). This indicates that EgLCY is a lycopene β-cyclase that converts lycopene to β-carotene.

### Effects of *EgLCY* Suppression on Cell Growth and Carotenoid Synthesis

**Figure 3:**
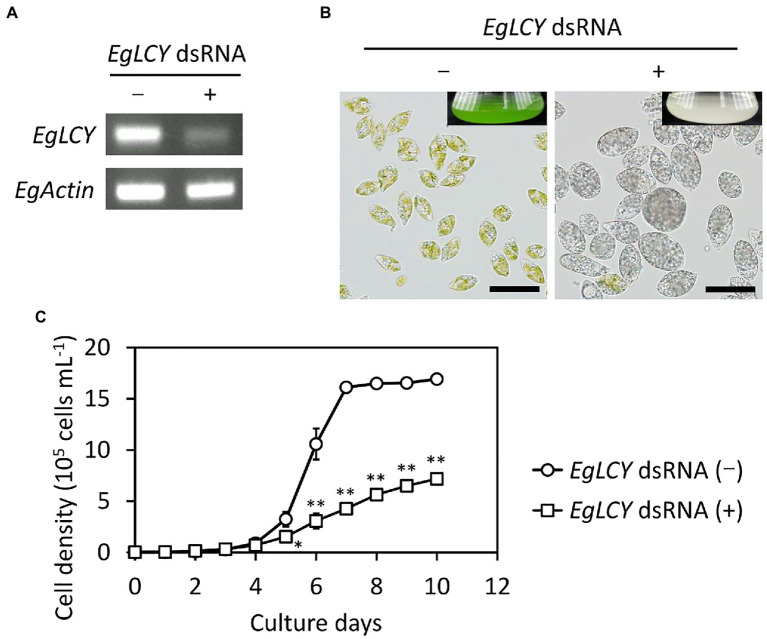
Effects of EgLCY suppression on cell appearance and growth. Cells treated with or without *EgLCY* dsRNA were mixotrophically grown under continuous light (40 μmol photons m^−2^ s^−1^) at 25°C. **(A)** *EgLCY* and *EgActin* transcript levels in cells treated with or without *EgLCY* dsRNA grown for 6 d. The *EgActin* gene was used as a constitutive control. **(B)** The appearances of cells and cultures (insets) of cells treated with or without *EgLCY* dsRNA grown for 6 d. Scale bars are 40 μm. **(C)** Growth curves of cells treated with or without *EgLCY* dsRNA grown for 10 d. Values are presented as the mean ± SD (*n* = 3). Values with asterisks are significantly different from cells treated without dsRNA according to the *t*-test (^*^p < 0.05; ^**^p < 0.01).

To examine the physiological role of EgLCY in *E. gracilis*, *EgLCY* knockdown cells were generated. Knockdown experiments were performed by introducing dsRNA of the *EgLCY* gene into *E. gracilis* cells by electroporation. *E. gracilis* cells electroporated without dsRNA showed the same carotenoid synthesis as wild-type cells (Kato et al., 2017). Semi-quantitative RT-PCR analysis indicated that *EgLCY* transcript levels were suppressed in *EgLCY* dsRNA-treated cells grown both mixotrophically (Figure 3A) and autotrophically (Supplementary Figure 3A) for 6 d. In contrast to the cells treated without dsRNA, both the *EgLCY* dsRNA-treated cells and culture medium were colorless. Moreover, the colorless *EgLCY* dsRNA-treated cells tended to adopt a hypertrophic morphology (Figure 3B). The cell densities of *EgLCY* dsRNA-treated cells grown mixotrophically decreased to 29% of cells treated without dsRNA after 6 d and 43% after 10 d (Figure 3C). The cell density of *EgLCY* dsRNA-treated cells grown autotrophically for 6 d also decreased significantly, whereas there was no significant difference after 10 days of growth (Supplementary Figure 3B). This may be due to the inability of *EgLCY* dsRNA-treated colorless cells to grow autotrophically and exclusive growth of a few green cells to which *EgLCY* dsRNA is not successfully introduced. These results suggest that EgLCY is functional *in vivo* and physiologically important for cell growth, regardless of the presence of a carbon source. Mixotrophic *EgLCY* dsRNA-treated cells tended to maintain longer phenotypic changes, and thus were used in subsequent analyses.

**Figure 4:**
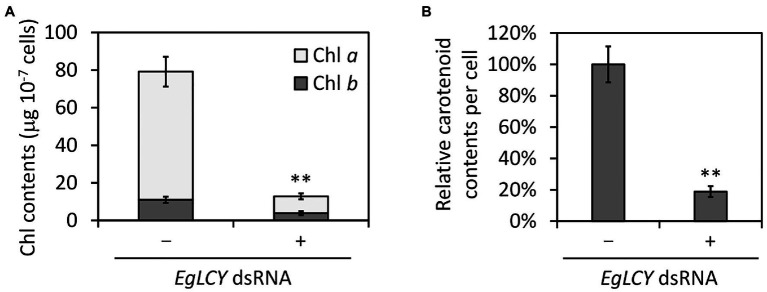
Effects of EgLCY suppression on pigment content. Cells treated with or without *EgLCY* dsRNA were mixotrophically grown under continuous light (40 μmol photons m^−2^ s^−1^) at 25°C for 6 d. **(A)** The chlorophyll (Chl) contents in cells treated with or without *EgLCY* dsRNA. **(B)** The relative total carotenoid contents of cells treated with or without *EgLCY* dsRNA. Values are presented as the mean ± SD (*n* = 3). Values with asterisks are significantly different from cells treated without dsRNA according to the *t*-test (^**^p < 0.01).

**Figure 5:**
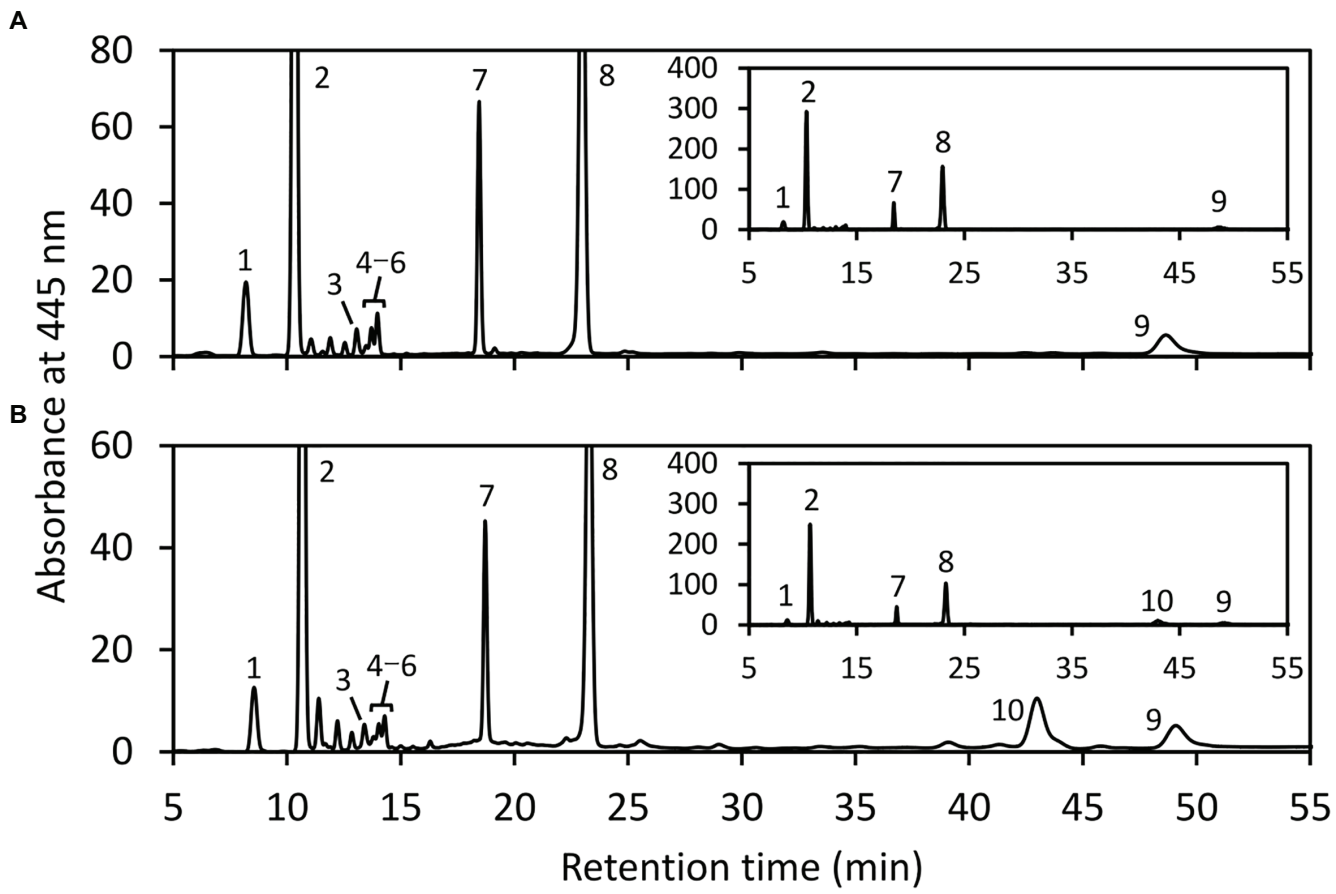
HPLC analysis of carotenoids extracted from *E. gracilis*. HPLC chromatogram (445 nm) of carotenoid extracts of cells treated without **(A)** or with *EgLCY* dsRNA **(B)** grown under continuous light (40 μmolm^−2^ s^−1^) at 25°C for 6 d. Insets show the same chromatograms with an expanded y axis. Higher number of cells treated with *EgLCY* dsRNA was used for carotenoid extraction to clearly detect each peak. The absorbance spectra of individual carotenoids and chlorophyll peaks were identical to our previous results (Tamaki et al., 2019) and those of β-carotene and lycopene in Figure 1. 1, neoxanthin; 2, diadinoxanthin; 3, all *trans*-diatoxanthin; 4–6, *cis*-diatoxanthins; 7, chlorophyll *b*; 8, chlorophyll *a*; 9, β-carotene; and 10, lycopene.

Next, we examined the chlorophyll and carotenoid content in *EgLCY* dsRNA-treated cells. The total chlorophyll content of the *EgLCY* dsRNA-treated cells decreased to 16% of the cells treated without dsRNA, in accordance with their appearance (Figure 4A). Carotenoid content and composition were analyzed using HPLC. In cells treated without dsRNA, diadinoxanthin, diatoxanthin, neoxanthin, and β-carotene were detected, whereas *EgLCY* dsRNA-treated cells contained additional lycopene, which is a substrate of EgLCY (Figure 5), further supporting the functional activity of EgLCY in *E. gracilis* cells. The total carotenoid content in *EgLCY* dsRNA-treated cells decreased to 19% of the cells treated without dsRNA (Figure 4B). β-carotene, neoxanthin, diadinoxanthin, and diatoxanthin were significantly decreased in *EgLCY* dsRNA-treated cells compared to cells treated without dsRNA, and lycopene accounted for 12% of the total carotenoid content in *EgLCY* dsRNA-treated cells (Table 1). These results indicate that EgLCY is essential for carotenoid synthesis in *E. gracilis*, and deficiencies in β-carotene and downstream carotenoid species, which are abundant and physiologically functional, suppress chlorophyll synthesis, resulting in colorless cells and probable loss of photosynthetic activity.

**Table 1:** Effects of *EgLCY* suppression on carotenoid compositions.

Carotenoid species	Relative carotenoid contents per cell (%)	
	*EgLCY* dsRNA (−)	*EgLCY* dsRNA (+)
β-carotene	8.2 ± 1.2	1.5 ± 0.3^**^
Neoxanthin	7.8 ± 1.2	1.0 ± 0.2^**^
Diadinoxanthin	77.5 ± 9.4	12.9 ± 2.7^**^
Ditoxanthin	6.5 ± 0.4	1.3 ± 0.2^**^
Lycopene	ND	2.3 ± 0.2

** *p* < 0.01.

Cells treated with or without EgLCY dsRNA were mixotrophically grown under continuous light (40 μmol photons m^−2^ s^−1^) at 25°C for 6 d. Each carotenoid content per cell is presented as the relative value when total carotenoid content in the cells treated without dsRNA is 100%. Values are presented as the mean ± SD (*n* = 3). Values with asterisks are significantly different from cells treated without dsRNA according to the *t*-test. ND, not detectable.

### Effects of *EgLCY* Suppression on the Ascorbate-Glutathione Cycle

**Figure 6:**
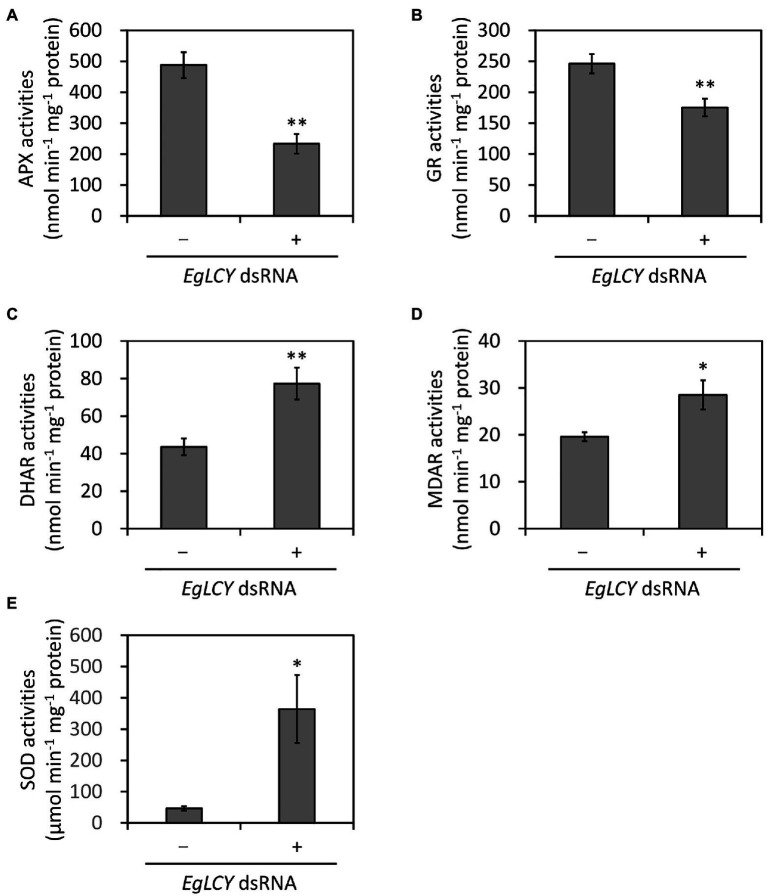
Effects of EgLCY suppression on the activities of the ascorbate-glutathione cycle enzymes. Cells treated with or without *EgLCY* dsRNA were mixotrophically grown under continuous light (40 μmol photons m^−2^ s^−1^) at 25°C for 6 d. Enzyme activities of ascorbate peroxidase **(A)**, glutathione reductase **(B)**, dehydroascorbate reductase **(C)**, monodehydroascorbate reductase **(D)**, and superoxide dismutase **(E)** in extracts from cells treated with or without *EgLCY* dsRNA. Values are presented as the mean ± SD (*n* = 3). Values with asterisks are significantly different from cells treated without dsRNA according to the *t*-test (^*^p < 0.05; ^**^p < 0.01).

To investigate the possible relationship between carotenoid synthesis and the ascorbate-glutathione cycle, the enzyme activities of APX, GR, DHAR, MDAR, and SOD in *EgLCY* dsRNA-treated cells were measured. The APX and GR activities of *EgLCY* dsRNA-treated cells were decreased to 48 and 71% of cells treated without dsRNA, respectively (Figures 6A,B). On the other hand, activity of DHAR and MDAR in *EgLCY* dsRNA-treated cells increased by 77 and 45%, respectively, compared to cells treated without dsRNA (Figures 6C,D). Moreover, the SOD activity of *EgLCY* dsRNA-treated cells increased 7.9 times compared to cells treated without dsRNA (Figure 6E). These results suggest the physiological importance of carotenoid synthesis for the upregulation of APX, GR, and SOD activities in *E. gracilis*.

To examine the effects on the gene expression of the ascorbate-glutathione cycle enzymes, semi-quantitative RT-PCR analysis was performed. Since the cDNA sequence of the *E. gracilis MDAR* gene is unknown (Ishikawa et al., 2017), *EgMDAR* was excluded from this analysis. As shown in Supplementary Figure 4, there was no significant difference in the transcript levels of *EgAPX*, *EgGR*, and *EgDHAR* genes between each treatment. This suggests that the ascorbate-glutathione cycle genes are post-transcriptionally regulated when β-carotene synthesis is inhibited.

**Figure 7:**
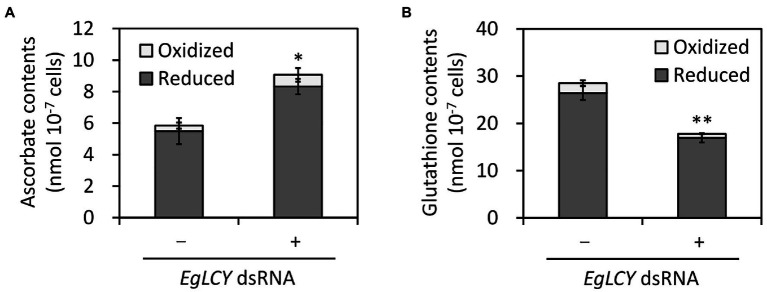
Effects of EgLCY suppression on antioxidant contents. Cells treated with or without *EgLCY* dsRNA were mixotrophically grown under continuous light (40 μmol photons m^−2^ s^−1^) at 25°C for 6 d. The contents of ascorbate **(A)** and glutathione **(B)** in extracts from cells treated with or without *EgLCY* dsRNA. Values are presented as the mean ± SD (*n* = 3). Values with asterisks are significantly different from cells treated without dsRNA according to the *t*-test (^*^p < 0.05; ^**^p < 0.01). Statistical analysis was performed on the total contents of each antioxidant.

It is presumed that significant changes in the activities of ascorbate-glutathione cycle enzymes are caused by the cellular levels of ascorbate and glutathione; therefore, the cellular content of these antioxidants was measured. The total ascorbate content of *EgLCY* dsRNA-treated cells was 55% higher than the cells treated without dsRNA (Figure 7A), while the total glutathione content of *EgLCY* dsRNA-treated cells was 62% less than the cells treated without dsRNA (Figure 7B). There was no significant difference in the redox ratios of both antioxidants between each treatment. This result clearly indicates that the size of the glutathione pool in *EgLCY* dsRNA-treated cells decreases with decreased GR activity.

### Effects of *EgLCY* Suppression on Cellular H_2_O_2_ Level

**Figure 8:**
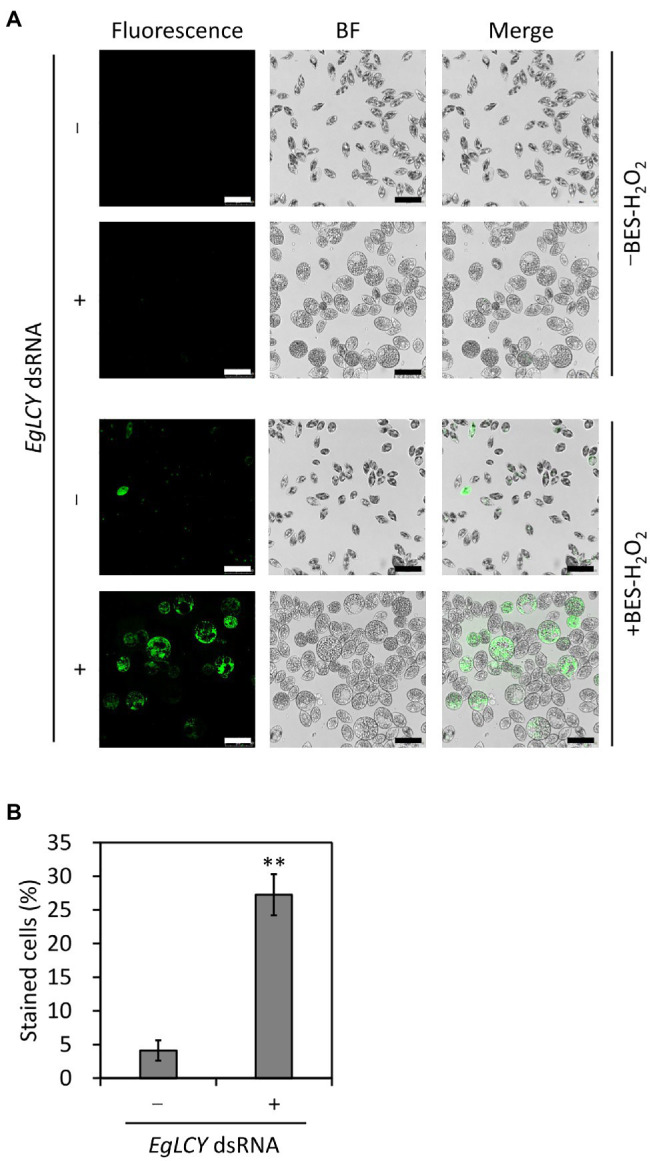
Effect of EgLCY suppression on cellular H_2_O_2_ levels. Cells treated with or without *EgLCY* dsRNA were mixotrophically grown under continuous light (40 μmol photons m^−2^ s^−1^) at 25°C for 6 d. **(A)** Fluorescent and bright field (BF) images of cells treated with or without *EgLCY* dsRNA. Cells were incubated with or without 20 μm BES-H_2_O_2_-Ac at room temperature for 30 min. Fluorescence was observed by confocal microscopy with a 485 nm laser and emission at 490–580 nm. Scale bars are 50 μm. **(B)** The staining rates of cells treated with or without *EgLCY* dsRNA. The criteria for staining are explained in Supplementary Figure 5. Values are presented as the mean ± SD (*n* = 3). More than 300 cells were included in each measurement. Values with asterisks are significantly different from cells treated without dsRNA according to the *t*-test (^**^p < 0.01).

To examine whether the decreases in APX activity and the size of the glutathione pool and increase in SOD activity due to *EgLCY* suppression affect oxidative stress status, the level of cellular H_2_O_2_ was visualized by staining with an H_2_O_2_-specific fluorescent reagent (BES-H_2_O_2_-Ac). As shown in Figure 8A, no fluorescence was observed in both treatments without BES-H_2_O_2_-Ac. When stained with BES-H_2_O_2_-Ac, fluorescence was detected in very few cells treated without dsRNA, whereas it was more apparent in *EgLCY* dsRNA-treated cells. A criterion for staining was defined based on the fluorescence intensity of each cell, as shown in Supplementary Figure 5, and this was used to calculate staining rates of each treatment. The staining rate of *EgLCY* dsRNA-treated cells was 27% and higher than that of cells treated without dsRNA (4%; Figure 8B), which suggests that *EgLCY* suppression causes a reduction in H_2_O_2_ scavenging capacity and results in the accumulation of oxidative stress in *E. gracilis*.

## Discussion

### Acquisition and Evolution of EgLCY in *E. gracilis*

*E. gracilis* is a euglenophyte species that has evolved independently of other photosynthetic organisms, although its strict phylogeny has yet to be fully elucidated. Genetic analysis suggests that two endosymbiotic events occurred; in the first, the euglenophyte acquired red lineage genes from Chromalveolata-like prey algae; in the second, green lineage genes from prasinophyte-like green algae were acquired (Maruyama et al., 2011). Since diadinoxanthin, the major carotenoid species of *E. gracilis*, is present in algae classified in Chromalveolata, such as diatoms and haptophytes, and not in green algae (reviewed in Tamaki et al., 2021), *E. gracilis* is believed to have acquired a series of carotenoid synthetic genes, including *LCY*, in the first endosymbiotic event. However, our analysis indicates that EgLCY is phylogenetically distinct from both diatom and green algae LCYs (Supplementary Figure 2). This may be due to the independent evolution of *E. gracilis*, as the Euglenozoa phylum diverged in the early stages of eukaryotic evolution (Ahmadinejad et al., 2007). The results of the phylogenetic analysis of β-carotene hydroxylase (CYP97H1; Tamaki et al., 2019) and ζ-carotene isomerase (Z-ISO; Sugiyama et al., 2020) correspond to those of EgLCY, suggesting that independent evolution of carotenoid synthetic genes in *E. gracilis* may be common.

An *in vivo* enzyme assay demonstrated that EgLCY catalyzes the cyclization of lycopene to β-carotene (Figure 2). RNAi-mediated suppression of *EgLCY* in *E. gracilis* caused bleaching, cell growth inhibition, and carotenoid and chlorophyll deficiencies (Figures 3, 4). Knockdown of other carotenoid synthetic genes, namely, *EgcrtB* and *EgCYP97H1*, also showed similar phenotypic changes (Kato et al., 2017; Tamaki et al., 2019), suggesting that EgLCY is the sole lycopene cyclase for β-carotene synthesis and for subsequent compounds, such as neoxanthin, diadinoxanthin, and diatoxanthin. *EgLCY* dsRNA-treated cells accumulated a small amount of lycopene, which is the substrate for EgLCY, and total carotenoid content in these cells was markedly decreased (Figures 4B, 5B and Table 1). As reported in our study of *EgCYP97H1*-suppressed cells, *E. gracilis* appears to regulate the whole of carotenoid synthesis in a manner that depends on the abundance of downstream carotenoid products, a regulatory system which differs from that of plants (Kim et al., 2009; Tamaki et al., 2019).

Plants and green algae have lycopene ε-cyclase, which catalyzes formation of the ε-ring of α-carotene and is necessary for the synthesis of abundant compound lutein (Supplementary Figure 2) (Cunningham et al., 1996). The lycopene ε-cyclase gene in *E. gracilis* was not identified by a homology search. This result supports the observation that *E. gracilis* lacks α-carotene and its derivatives (Figure 5) and that EgLCY produces β-carotene (Figure 2). The absence of lycopene ε-cyclase is consistent with algae classified in Chromalveolata, including diatoms that produce diadinoxanthin (Dambek et al., 2012).

### Proposed Relationship Between Carotenoid Synthesis and the Ascorbate-Glutathione Cycle Against H_2_O_2_ Accumulation

Carotenoids play an important role in photoprotection by receiving excess energy from photoexcited chlorophyll and scavenging singlet oxygen (reviewed in Tamaki et al., 2021). The inhibited synthesis of β-carotene and downstream carotenoid species by *EgLCY* suppression would accumulate excess photoexcited chlorophyll and subsequent singlet oxygen and superoxide radical, and cause damage to photosynthetic apparatus and chloroplast dedifferentiation (Cazzaniga et al., 2012; Santabarbara et al., 2013). Carotenoid deficiency-induced impairment of chloroplast development and inhibited chlorophyll synthesis was agreed with previous study using *E. gracilis* treated with norflurazon (Madhusudhan et al., 2003) and knocked down cells of carotenoid synthetic genes *EgcrtB* (Kato et al., 2017) and *EgCYP97H1* (Tamaki et al., 2019). In the *EgLCY* dsRNA-treated cells, increase in the SOD activity rapidly converts superoxide radical to H_2_O_2_ (Figure 6E), which then diffuses into the cytosol from chloroplasts (Ishikawa et al., 1993). *E. gracilis* APX enzyme is localized in the cytosol and is post-transcriptionally photo-induced by retrograde signal from chloroplasts (Madhusudhan et al., 2003; Ishikawa et al., 2010). Chloroplast dedifferentiation in *EgLCY* dsRNA-treated cells would stagnates retrograde signaling and inhibits APX photoinduction. The glutathione pool size is speculated to be regulated by a similar mechanism based on the fact that the glutathione pool size is decreased in chloroplast-dedifferentiated SM-ZK strain of *E. gracilis* in which retrograde signals from plastids are suppressed (Shigeoka et al., 1987b). As a consequence of them, it can be explained that *EgLCY* suppression causes downregulation of APX activity and decreased glutathione pool size (Figures 6A,B, 7B). In *E. gracilis* lacking catalase, since APX is a major H_2_O_2_ scavenging enzyme, downregulation of APX activity due to the inhibited synthesis of β-carotene and downstream carotenoid species excessively increases cellular H_2_O_2_ level (Figure 8). Although *EgLCY* suppression increased ascorbate pool size (Figures 6C,D, 7A), it is speculated that excess H_2_O_2_ accumulation cannot be suppressed because the ROS scavenging capacity of ascorbate itself is extremely lower than that of APX (Smirnoff and Arnaud, 2019).

Both carotenoids and the ascorbate-glutathione cycle are physiologically essential for ROS scavenging in photosynthetic organisms, including *A. thaliana* and *Chlamydomonas reinhardtii*; however, the understanding of the possible relationship between carotenoid synthesis and the ascorbate-glutathione cycle components in other photosynthetic organisms is currently limited. Ascorbate-deficient *A. thaliana* mutants showed decreased ascorbate content and poor growth but no difference in carotenoid and chlorophyll content compared to WT (Plumb et al., 2018). Norflurazon is an herbicide that inhibits phytoene desaturase, resulting in the depletion of carotenoids, and is often used in carotenoid research in photosynthetic organisms. Treatment of *A. thaliana* and cucumber (*Cucumis sativus*) with norflurazon had little effect on the activities of APX and GR (Jung et al., 2000; Jung, 2004). Moreover, norflurazon treatment of *A. thaliana* and rice did not affect ROS (superoxide radical, H_2_O_2_, and singlet oxygen) accumulation and lipid peroxidation (Kim and Apel, 2013; Park and Jung, 2018). In the green alga *C. reinhardtii*, APX has been reported to be important for resistance to photooxidative stress, in cooperation with ascorbate recycling by DHAR and MDAR (Kuo et al., 2020). Carotenoid-deficient *C. reinhardtii* generated by either a phytoene synthase mutation (*lts1-204*) or norflurazon treatment showed high levels of oxidative stress; however, the effect of carotenoid deficiency on the ascorbate-glutathione cycle enzymes was not examined (Pérez-Pérez et al., 2012). Our study clearly explained that EgLCY-mediated synthesis of β-carotene and downstream carotenoid species caused upregulated APX activity, increased glutathione pool, and decreased cellular H_2_O_2_ accumulation (Figures 6–8). This suggests a possible relationship between carotenoid synthesis and the ascorbate-glutathione cycle for ROS scavenging in *E. gracilis*.

### Distinct Regulation of Ascorbate and Glutathione Synthesis in *E. gracilis*

In many photosynthetic organisms, ascorbate and glutathione share similar functions and act cooperatively as components of the ascorbate-glutathione cycle. Previous studies reported that ascorbate and glutathione were synthesized in *E. gracilis* by exposure to blue light (wavelengths from 380 nm to 440 nm) and are not associated with photosynthesis under mixotrophic conditions (Shigeoka et al., 1979, 1987c). Therefore, it is expected that these antioxidants can be synthesized synchronously. In the green alga *Dunaliella salina*, both cold and high light treatment decreased amounts of β-carotene and chlorophyll, while ascorbate and glutathione increased, suggesting that the oxidative stress caused by pigment deficiencies was alleviated by the induction of both ascorbate and glutathione synthesis (Haghjou et al., 2009). If *E. gracilis* has a similar regulatory mechanism for ascorbate and glutathione synthesis, the content of these compounds would increase when carotenoid and chlorophyll synthesis are suppressed. However, this study unexpectedly revealed that ascorbate and glutathione synthesis, and their recycling enzyme activities, were different in *EgLCY* dsRNA-treated cells (Figures 6B–D, 7). Promotion of ascorbate synthesis in *EgLCY* dsRNA-treated cells is likely to be one of the mechanisms responsible for the toxic effects of H_2_O_2_ accumulation to avoid stagnation of the entire ascorbate-glutathione cycle. In contrast, we also observed that glutathione synthesis was triggered by carotenoid accumulation, which suggests that ascorbate and glutathione synthesis are differentially regulated in *E. gracilis* under oxidative stress conditions. *E. gracilis* may initially synthesize ascorbate rather than glutathione to cope with accumulated oxidative stress.

## Conclusion

In this study, we identified and functionally characterized EgLCY; an *in vivo* enzyme assay demonstrated the lycopene β-cyclase activity of EgLCY. Knocking down *EgLCY* in *E. gracilis* resulted in colorless cell with hypertrophic morphologies along with growth inhibition and reduced carotenoid and chlorophyll synthesis. Moreover, *EgLCY* suppression affected the ascorbate-glutathione cycle by reducing APX activity and decreasing the glutathione pool size, while the ascorbate pool size and SOD activity increased. As a consequence of *EgLCY* suppression and its associated effects mentioned above, *E. gracilis* accumulated high levels of H_2_O_2_, which was scavenged mainly by APX. These results suggest that the synthesis of β-carotene and downstream carotenoids is a physiologically important for defense against H_2_O_2_ accumulation by upregulating APX activity and the glutathione pool. To our knowledge, this is the first evidence of a possible relationship between carotenoid synthesis and the ascorbate-glutathione cycle for ROS scavenging in photosynthetic organisms.

## Data Availability Statement

The datasets presented in this study can be found in online repositories. The names of the repository/repositories and accession number(s) can be found in the article/Supplementary Material.

## Author Contributions

ST and TS conceived the research plans. ST, RS, and YKos performed the experiments. MA, YKod, TI, and TS supervised the experiments. ST, RS, and TS designed the experiments and analyzed the data. ST wrote the article. All authors contributed to the article and approved the submitted version.

## Funding

This work was supported by the Japan Society for the Promotion of Science Grant-in-Aid for Scientific Research (grant number 17K07945 to TS) and the Sasakawa Scientific Research Grant from the Japan Science Society (grant number 2020–4050 to ST).

## Conflict of Interest

The authors declare that the research was conducted in the absence of any commercial or financial relationships that could be construed as a potential conflict of interest.

## Publisher’s Note

All claims expressed in this article are solely those of the authors and do not necessarily represent those of their affiliated organizations, or those of the publisher, the editors and the reviewers. Any product that may be evaluated in this article, or claim that may be made by its manufacturer, is not guaranteed or endorsed by the publisher.
