# Supplementary material for: Suppression of the Lycopene Cyclase Gene Causes Downregulation of Ascorbate Peroxidase Activity and Decreased Glutathione Pool Size, Leading to H2O2 Accumulation in Euglena gracilis
Source: Front Plant Sci. 2021 Dec 3;12:786208. doi: 10.3389/fpls.2021.786208 (PMC8678482; doi:10.3389/fpls.2021.786208)
Supplement: Supplementary file 1 [file Data_Sheet_1.pdf]

```
EgLCY           TVARYQNTELPQYVPS--HTYAAAVVGGGPAGLALAWNLAKRGVSVVVVDNV-IEKHWPN 237
AtLCYB          TKKENLDFELPLYDTSKSQVVDLAIVGGGPAGLAVAQQVSEAGLSVCSIDPS-PKLIWPN 121
SlLCYB          TKKENLDFELPMYDPSKGVVVDLAVVGGGPAGLAVAQQVSEAGLSVCSIDPN-PKLIWPN 122
NtLCYB          TKKENLDFELPMYDPSKGLVVDLAVVGGGPAGLAVAQQVSEAGLSVVSIDPS-PKLIWPN 122
Syn7942CrtL     -------------------MFDALVIGSGPAGLAIAAELAQRGLKVQGLSPVDPFHPWEN 41
                                        ::*.******:* :::: *:.*  :.       * *

EgLCY           NYGVWLDEWDALGFPEATLGIRWEYTTIRYTETEATRLPRGYGRVDRKVLKEYLLAQCVK 297
AtLCYB          NYGVWVDEFEAMDLLDC-LDTTWSGAVVYVDEGVKKDLSRPYGRVNRKQLKSKMLQKCIT 180
SlLCYB          NYGVWVDEFEAMDLLDC-LDATWSGAAVYIDDNTAKDLHRPYGRVNRKQLKSKMMQKCIM 181
NtLCYB          NYGVWVDEFEAMDLLDC-LDATWSGTVVYIDDNTTKDLDRPYGRVNRKQLKSKMMQKCIL 181
Syn7942CrtL     TYGIWGPELDSLGLEHL-FGHRWSNCVSYFGEAPVQHQYN-YGLFDRAQLQQHWLRQCEQ 99
                .**:*  * :::.: .  :.  *.  .    :       . ** .:*  *:.  : :*

EgLCY           CGVHFAAGSAASITEGAGEESSIVNLVGGQSVTCKLPVVAAGHYSKLIKYHSPGEKFVNS 357
AtLCYB          NGVKFHQSKVTNVVHEE--ANSTVVCSDGVKIQASVVLDATGFSRCLVQYDKP------- 231
SlLCYB          NGVKFHQAKVIKVIHEE--SKSMLICNDGITIQATVVLDATGFSRSLVQYDKP------- 232
NtLCYB          NGVKFHHAKVIKVIHEE--AKSMLICNDGVTIQATVVLDATGFSRCLVQYDKP------- 232
Syn7942CrtL     GGLQWQLGKAAAIAHDS--HHSCVTTAAGQELQARLVVDTTGHQAAFIQRPHS------- 150
                 *:::  ...  : .      * :    *  : . : : ::*.   :::   .

EgLCY           KGEYMTQNWGELVAEKNGPNPWGWQFSQGGAPGYQIAYGIEVETDGPHGFPLNEMVLMDW 417
AtLCYB          -----------------------------YNPGYQVAYGIVAEVD-GHPFDVDKMVFMDW 261
SlLCYB          -----------------------------YNPGYQVAYGILAEVE-EHPFDVNKMVFMDW 262
NtLCYB          -----------------------------YKPGYQVAYGILAEVE-EHPFDTSKMVLMDW 262
Syn7942CrtL     -----------------------------DAIAYQAAYGIIGQFS-QPPIEPHQFVLMDY 180
                                                .** ****  : .    :   ::*:**:

EgLCY           SGEHVQGREKEAQWTG-SPTFLYVMPTDETHAFLEETSLVGRPAITVADCKERLAVRLQH 476
AtLCYB          RDKHLDSYPELKERNSKIPTFLYAMPFSSNRIFLEETSLVARPGLRMEDIQERMAARLKH 321
SlLCYB          RDSHLKNNTDLKERNSRIPTFLYAMPFSSNRIFLEETSLVARPGLRIDDIQERMVARLNH 322
NtLCYB          RDSHLGNNMELKERNRKVPTFLYAMPFSSNKIFLEETSLVARPGLRMDDIQERMVARLNH 322
Syn7942CrtL     RSDHLS-----PEERQLPPTFLYAMDLGNDVYFVEETSLAACPAIPYDRLKQRLYQRLAT 235
                 ..*:       :     *****.*  ..   *:*****.. *.:     ::*:  **

EgLCY           RGIRVKKILEAESCVIPMGGPLPILGGRSVPYGASNNLVHPATGYMVNRAIGNSAKVADA 536
AtLCYB          LGINVKRIEEDERCVIPMGGPLPVLPQRVVGIGGTAGMVHPSTGYMVARTLAAAPIVANA 381
SlLCYB          LGIKVKSIEEDEHCLIPMGGPLPVLPQRVVGIGGTAGMVHPSTGYMVARTLAAAPVVANA 382
NtLCYB          LGIKVKSIEEDEHCVIPMGGSLPVIPQRVVGTGGTAGLVHPSTGYMVARTLAAAPVVANA 382
Syn7942CrtL     RGVTVQVIQHEEYCLFPMNLPLPDLTQSVVGFGGAASMVHPASGYMVGALLRRAPDLANA 295
                 *: *: * . * *::**. .** :    *  *.: .:***::****   :  :. :*:*

EgLCY           VATALEAG----RSLPAVTAAAWDAIWPQEALRVRDFQVFGMEVLLNMDLNQCRDFFKTF 592
AtLCYB          IVRYLGSPSSNSLRGDQLSAEVWRDLWPIERRRQREFFCFGMDILLKLDLDATRRFFDAF 441
SlLCYB          IIQYLGS--ERSHSGNELSTAVWKDLWPIERRRQREFFCFGMDILLKLDLPATRRFFDAF 440
NtLCYB          IIHYLGS--EKDLLGNELSAAVWKDLWPIERRRQREFFCFGMDILLKLDLPATRRFFDAF 440
Syn7942CrtL     IAAGLNA--SSSLTTAELATQAWRGLWPTEKIRKHYIYQFGLEKLMRFSEAQLNHHFQTF 353
                :   * :          ::: .*  :** *  * : :  **:: *:.:.    . .*.:*
```

Supplemental Figure 1. Sequence alignment of LCY proteins in *E. gracilis* and other organisms. Amino acid sequence alignment was constructed using the ClustalW program. The FAD/NADPH binding site and cyclase motifs are highlighted in light and dark gray, respectively. Asterisks indicate the amino acids conserved in all sequences, and colons and dots indicate amino acids with similar biochemical characteristics. GenBank accession numbers for the LCY orthologs are as follows: *Arabidopsis thaliana* (AtLCYB: U50739); *Solanum lycopersicum* (SlLCYB: X86452); *Nicotiana tabacum* (NtLCYB: X81787); *Synechococcus elongatus* PCC 7942 (Syn7942CrtL: X74599).

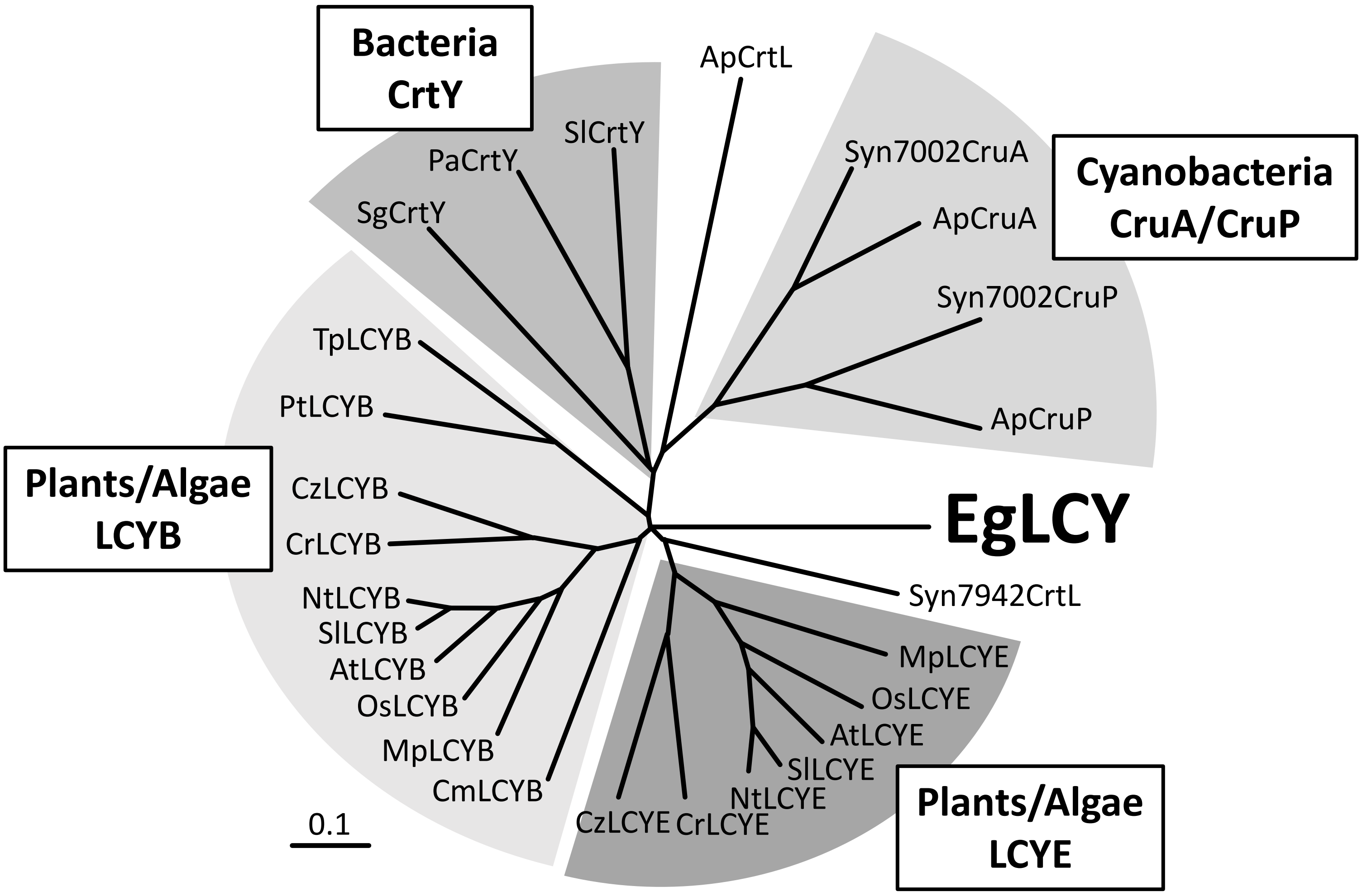

Supplemental Figure 2. Phylogenetic tree of LCY proteins in *E. gracilis* and other organisms. A phylogenetic tree was constructed using the ClustalW program and visualized with TreeView. GenBank accession numbers for the LCY orthologs are as follows: *Arabidopsis thaliana* (AtLCYB: U50739; AtLCYE: U50738); *Oryza sativa* (OsLCYB: AP014958; OsLCYE: AK066182); *Solanum lycopersicum* (SlLCYB: X86452; SlLCYE: Y14387); *Nicotiana tabacum* (NtLCYB: X81787; NtLCYE: KC484707); *Marchantia polymorpha* (MpLCYB: AB794089; MpLCYE: AB794090); *Synechococcus* sp. PCC 7002 (Syn7002CruA: EF529626; Syn7002CruP: EF529627); *Synechococcus elongatus* PCC 7942 (Syn7942CrtL: X74599); *Arthrospira platensis* (ApCrtL: AP011615; ApCruA: AP011615; ApCruP: AP011615); *Chlamydomonas reinhardtii* (CrLCYB: AY860818; CrLCYE: DS496110); *Chromochloris zofingiensis* (CzLCYB: FN563998; CzLCYE: HE664109); *Cyanidioschyzon merolae* (CmLCYB: AP006493); *Phaeodactylum tricornutum* (PtLCYB: MG397136); *Thalassiosira pseudonana* (TpLCYB: CM000639); *Pantoea ananas* (PaCrtY: D90087); *Sphingomonas lacus* (SlCrtY: KF724895); *Streptomyces griseus* (SgCrtY: AF272737).

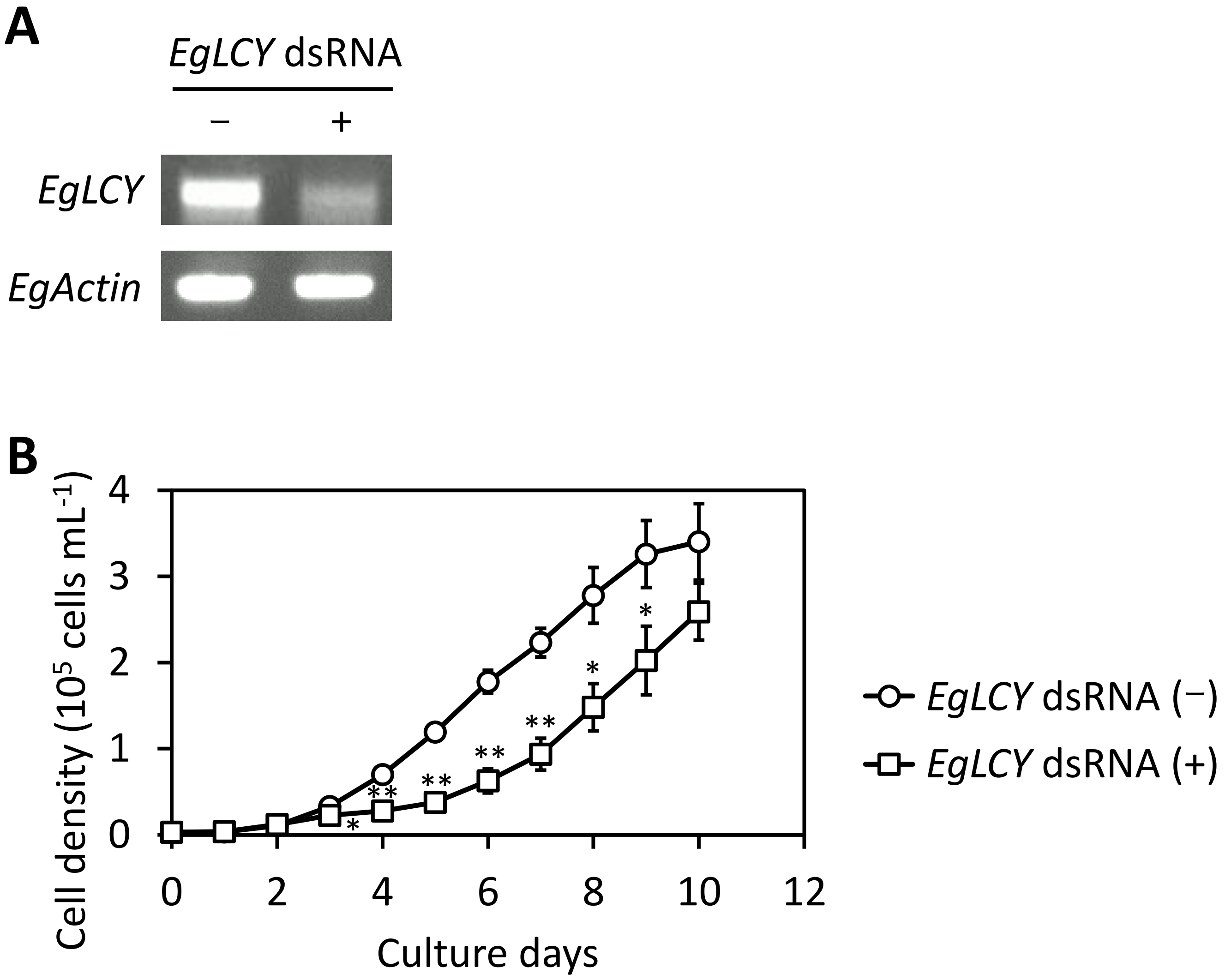

Supplemental Figure 3. Effect of EgLCY suppression on autotrophic cell growth in *E. gracilis*. Cells treated with or without *EgLCY* dsRNA were autotrophically grown under continuous light (40 μmol photons m^{-2} s^{-1}) at 25°C. (A) *EgLCY* and *EgActin* transcript levels in cells treated with or without *EgLCY* dsRNA grown for 6 d. Semi-quantitative RT-PCR was performed using specific primers for the *EgLCY* and *EgActin* genes with cDNA prepared from cells treated with or without *EgLCY* dsRNA. The *EgActin* gene was used as a constitutive control. (B) Growth curves of cells treated with or without *EgLCY* dsRNA grown for 10 d. Values are presented as the mean ± SD (n=3). Values with asterisks are significantly different from cells treated without dsRNA according to the t-test (*, p<0.05; **, p<0.01).

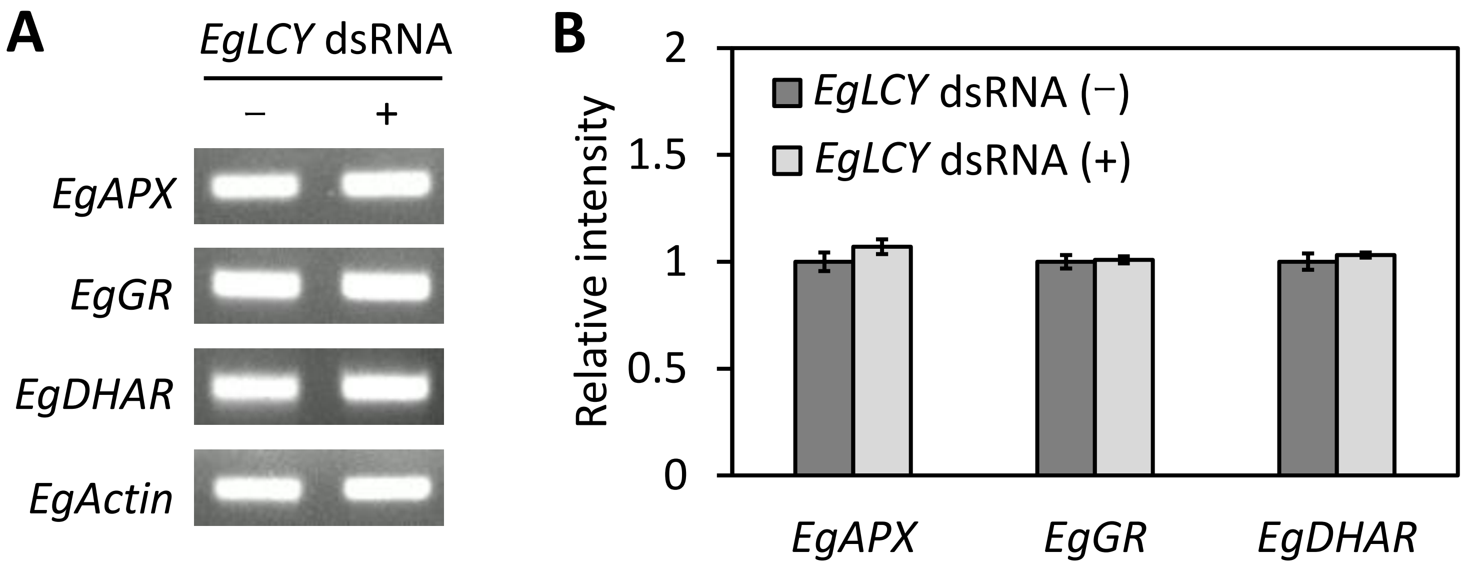

Supplemental Figure 4. Semi-quantitative RT-PCR analysis of ascorbate-glutathione cycle genes in cells treated with or without *EgLCY* dsRNA. Cells were grown under continuous light (40 μmol photons m^{-2} s^{-1}) at 25 °C for 6 d. (A) Electrophoresis image of semi-quantitative RT-PCR product. PCR was performed using specific primers for the *EgAPX*, *EgGR*, *EgDHAR*, and *EgActin* genes, with cDNA prepared from each cell line. The *EgActin* gene was used as a constitutive control. (B) Relative band intensities of each gene quantified using ImageJ (https://imagej.nih.gov/ij/). Relative intensity indicates the value obtained by normalizing the transcript level of each gene with that of *EgActin*. Values are presented as the mean ± SD (*n*=3). There was no significant difference.

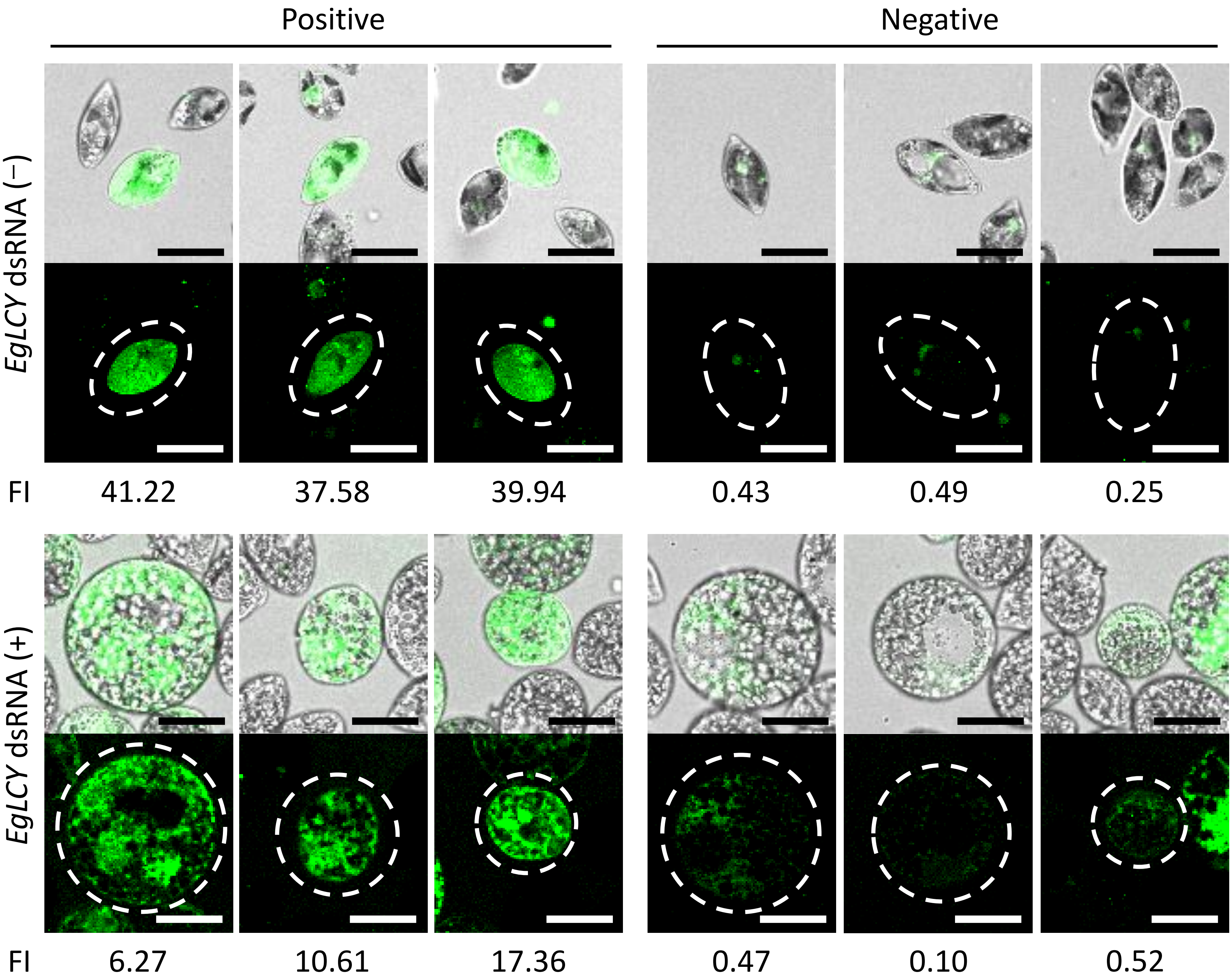

Supplemental Figure 5. Quantification of fluorescence intensities obtained from fluorescent images of cells treated with or without *EgLCY* dsRNA and then stained with BES-H_2O_2-Ac. Images are representative examples of each experimental condition. The upper panels are merged brightfield and fluorescence images, and the lower panels are fluorescent images. The values shown below each image indicate the fluorescence intensities per cell quantified by ImageJ (https://imagej.nih.gov/ij/). Positive and negative cells were defined as fluorescence intensity exceeding 1. Scale bars are 20 μm. FI, fluorescence intensity (arbitrary units).

Supplemental Table 1. Primers used in this study.

Primer name	Sequence
EgLCY-F	5’-ATCATGGCACATCATGCTG-3’
EgLCY-R	5’-TCACAGGTTCAGCAGTGACTTC-3’
EgLCY-InFusion-F	5’-AGGAGATATACCATGGCACATCATGCTGTGCATC-3’
EgLCY-InFusion-R	5’-TGGCTGCTGCCCATGTCACAGGTTCAGCAGTGAC-3’
EgLCY-RNAi-F	5’-TAATACGACTCACTATAGGGAAATGCAAACTTCCAGCATGC-3’
EgLCY-RNAi-R	5’-TAATACGACTCACTATAGGGCAGTGCTTCTCGATGACGTTG-3’
EgLCY-sqRT-PCR-F	5’-AAATGCAAACTTCCAGCATGC-3’
EgLCY-sqRT-PCR-R	5’-CAGTGCTTCTCGATGACGTTG-3’
EgActin-sqRT-PCR-F	5’-CAAGAGCAATCGTGAGAAGATG-3’
EgActin-sqRT-PCR-R	5’-CACCAGACAGCACAATGTTG-3’
EgAPX-sqRT-PCR-F	5’-GACGAGGAGATCGTGGC-3’
EgAPX-sqRT-PCR-R	5’-AAACACGGCCTGGAAAGATATG-3’
EgGR-sqRT-PCR-F	5’-ATGACCTTCATTATGCGAAGTC-3’
EgGR-sqRT-PCR-R	5’-CATTTCCGTGCAGAGATGG-3’
EgDHAR-sqRT-PCR-F	5’-AAGTGGAGTTAAGAGCTGTCGG-3’
EgDHAR-sqRT-PCR-R	5’-AGCCATATCCACTTTCTGCAAAG-3’

T7 RNA polymerase promoter sequences are underlined.
